# Laparoscopic management of gastric remnant ischemia after laparoscopic distal gastrectomy with Billroth-I anastomosis—A case report

**DOI:** 10.1016/j.ijscr.2019.12.009

**Published:** 2019-12-13

**Authors:** Abdulaziz Alshehri, Hee-sung Kim, Byung Sik Kim

**Affiliations:** a Department of Gastric Surgery, Ulsan University School of Medicine, Asan Medical Center, Seoul, Republic of Korea; b General Surgery Department, King Fahad Military Medical Complex, Dhahran, Saudi Arabia

**Keywords:** Gastric remnant, Ischemia, Laparoscopic surgery, Esophagogastroduodenoscopy, Subtotal gastrectomy, Anastomosis, Roux-en-Y

## Abstract

**Introduction:**

Gastric remnant ischemia after laparoscopic distal gastrectomy (LDG) in gastric cancer patients is a very rare but life-threatening condition, especially when accompanied by a splenic infarction or unplanned splenectomy.

**Presentation of case:**

A 72-year-old male with no comorbid diseases was diagnosed with a well-differentiated adenocarcinoma of the lower stomach and underwent LDG with D2 lymph node dissection. However, a splenic artery injury necessitated a splenectomy intra-operatively, and delta anastomosis was performed based on a clinically viable remnant stomach. During the late post-operative period, the patient developed abdominal pain and showed increased levels of inflammatory biomarkers with hemodynamic stability. Esophagogastroduodenoscopy (EGD) showed necrotic patches over the distal part of the remnant stomach with normal anastomosis and duodenal mucosa. Progression of the necrosis was noted on follow up EGD performed 15 days post-surgery. Total laparoscopic subtotal gastrectomy with Roux-en-Y reconstruction was performed and the patient recovered uneventfully.

**Discussion:**

Careful dissection of the lymph nodes over the major vessels is essential to avoid complications in gastric cancer patients requiring LDG. Moreover, major complications occurring intra-operatively may necessitate changes in the surgical plan, including re-excision of the remnant stomach or conversion to Roux-en-Y reconstruction.

**Conclusion:**

Careful evaluation of the clinical findings and close observation with EGD can help detect mucosal demarcation lines and ascertain the perfect timing for intervention in cases with suspected ischemia. Although gastric remnant ischemia requires emergency treatment, laparoscopic management is a feasible option when performed by an expert surgeon.

## Highlights

• Gastric remnant ischemia after laparoscopic distal gastrectomy (LDG) is very rare.
• Laparoscopic subtotal gastrectomy for gastric remnant ischemia is effective.
• Routine esophagogastroduodenoscopy and laparoscopic can help avoid fatal outcomes.

## 1 Introduction

Good gastric cancer screening programs in South Korea are attributable for the high rate of early gastric cancer detection in this region, which has in turn led to the increasing use of laparoscopic gastrectomy and lymph node dissection including that of D1, D1+, and D2 lymph nodes with a variety of reconstruction techniques in this field with an excellent reported survival rate [1]. Gastric remnant ischemia is one of the very rare complications of this procedure and can occur in certain complicated cases especially those complicated intra-operatively by splenic infarction or iatrogenic vascular injury mandating splenectomy [2,3].

Here, we present a case wherein postoperative ischemia was detected in a patient after laparoscopic distal gastrectomy (LDG) during which splenectomy was performed owing to splenic artery injury. Close follow-up with esophagogastroduodenoscopy (EGD) and laparoscopic exploration helped us avoid fatal consequences.

This work has been reported in line with the SCARE criteria [4].

## 2 Presentation of case

A 72-year-old male with no comorbid diseases was admitted after routine gastric screening with an endoscopic diagnoses of type IIc lower stomach cancer. Endoscopic ultrasound showed a lesion extending to the submucosal layer (T1, Nx, Mx). Histopathological examination showed early well-differentiated gastric adenocarcinoma (type IIc). All blood parameters and tumour markers were within normal limits.

**Fig. 1:**
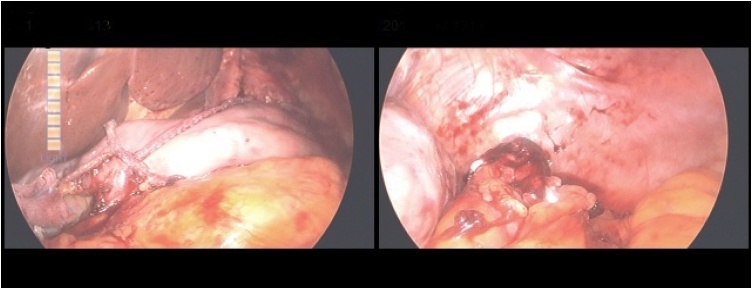
Intra-operative image of the delta anastomosis during the first surgery.

Abdominal computed tomography (CT) with contrast showed no demonstratable primary gastric lesion or metastatic lesions; however, an enlarged lymph node (LN) in the lesser curvature side was noted. LDG with D2 LN dissection was performed. However, an iatrogenic splenic artery injury occurred intraoperatively during LN dissection mandating splenectomy. Billroth-I reconstruction was performed after confirming the clinical viability of the gastric remnant and a drain (*J*-Vac) was inserted (Fig. 1). The histological examination of the final specimen revealed T1b, N1, M0.

**Fig. 2:**
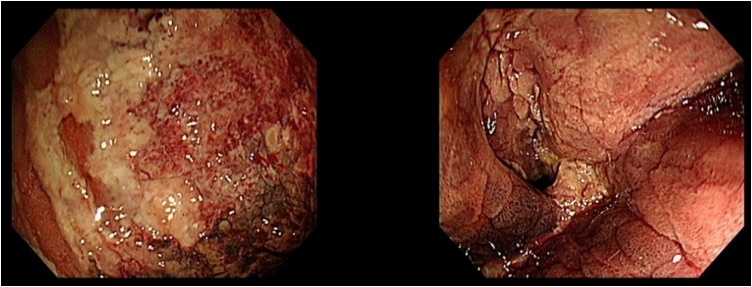
Day 3 postoperative esophagogastroduodenoscopy showing a layer of exudate covering the hyperaemic gastric mucosa.

Day 2 postoperatively, upper abdominal pain was noticed by the patient; however, all other clinical signs were normal with a slight increase in laboratory parameters, including complete blood count and C-reactive protein (CRP) levels, within the normal range. Over the next two days, the abdominal pain persisted, and the patient developed fever (38.3 ℃). Heart rate was 84/min with significant elevation of inflammatory markers (white blood cell [WBC], 21.9 × 10^3^/μL; CRP, 26.88 mg/dl; Table 1). Esophagogastroduodenoscopy (EGD) revealed distal remnant stomach necrotic and oedematous changes but the anastomosis site and duodenal mucosa were normal (Fig. 2).

**Table 1:** Clinical and laboratory findings during postoperative follow-up.

	Day 2	Day 5	Day 7	Day 10	Day 13	Day 17	Day 19
Fever (℃)	37.6	38.3	36.5	36.4	36.2	36.6	38.1
Heart rate (/min)	89	84	82	75	83	79	105
WBC (×10^3^/μL)	12.8	21.9	17.0	11.1	11.7	11.9	14.1
CRP (mg/dl)	4.7	26.88	10.7	5.22	4.24	4.47	23.4

WBC, White blood cell; CRP, C-reactive protein.

Conservative treatment involved nil per os and administration of total parenteral nutrition was initiated with close observation. Subsequently, the patient’s clinical condition improved with no fever and very mild intermittent upper abdominal pain. Moreover, his laboratory findings improved (WBC 11.1 × 10^3^/μL; CRP 5.22 mg/dl).

**Fig. 3:**
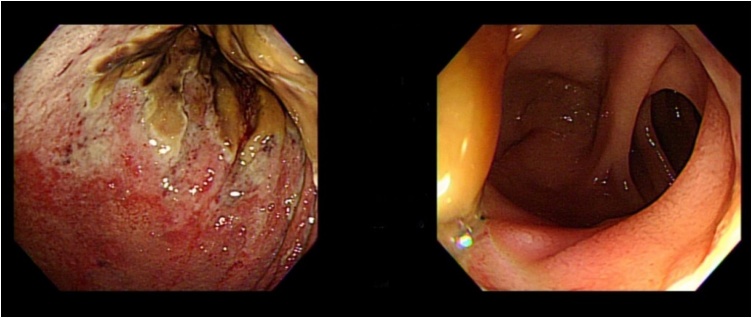
Day 7 postoperative esophagogastroduodenoscopy, (left) showing demarcating necrotic patches over the gastric mucosa and (right) intact healthy gastro-duodenal anastomosis.

On postoperative day 10, follow-up EGD (Fig. 3) showed that the mucosal necrosis had improved slightly along with other clinical parameters; thus, ingestion of oral fluid was allowed and tolerated well by the patient. No fever was noted. On postoperative day 15, the patient again developed upper abdominal pain with high fever. His laboratory parameters were also elevated (WBC 19.4 × 10^3^/μL; CRP 6.55 mg/dl; Table 1).

**Fig. 4:**
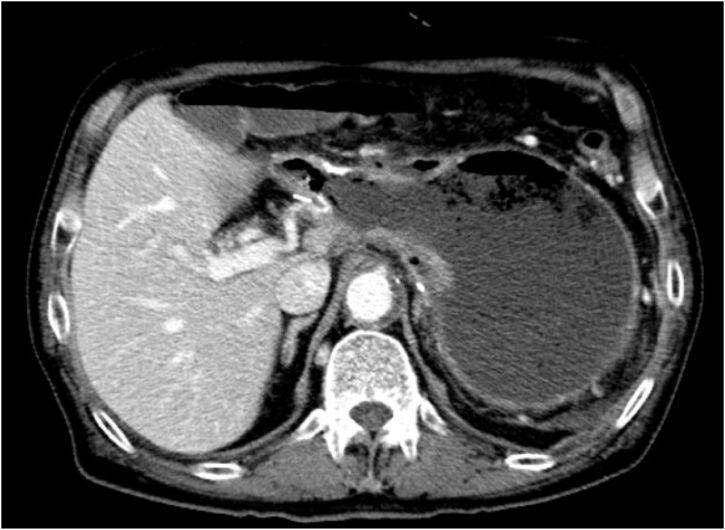
Axial computed tomography scan of the abdomen suggesting a high possibility of gastric ischemia and some fluid around the stomach.

**Fig. 5:**
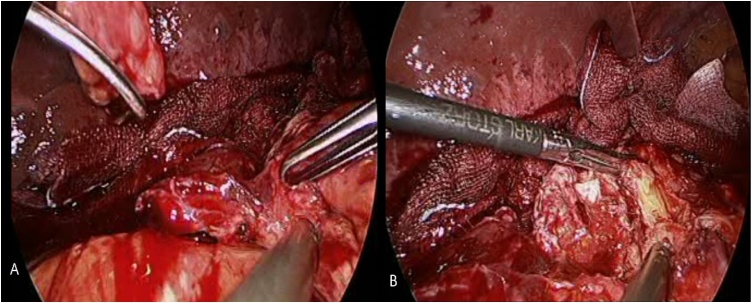
Laparoscopic exploration showing A- intact gastroduodenal anastomosis, and B- ischemic and necrotic portions in the distal part of the remnant stomach.

**Fig. 6:**
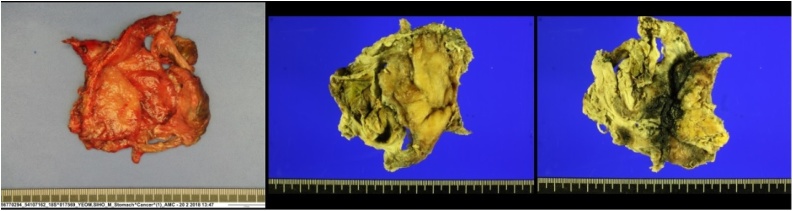
Histopathological gross photo of re-excised ischemic stomach part.

EGD showed that the necrosis had worsened, and progressive ischemia was suspected. Abdominal CT with contrast also suggested a high possibility of gastric ischemia (Fig. 4). Subsequently, laparoscopic exploration was performed and necrosis of the distal part of the remnant stomach was noted with severe adhesion over it (Fig. 5, Fig. 6).

Subtotal gastrectomy leaving approximately 10 % of the proximal viable stomach was performed with Roux-en-Y gastrojejunostomy reconstruction. Two large J-vac drains were inserted, one under the gastrojejunostomy site and one near the resected duodenal stump. Postoperatively, the patient was stable and had an uneventful hospital course. He was started on oral fluids on the second day post-operatively with no complications. On postoperative day 5, all clinical and laboratory markers had dramatically improved (WBC 10.1 × 10^3^/μL; CRP 6.1 mg/dl; Table 1).

**Fig. 7:**
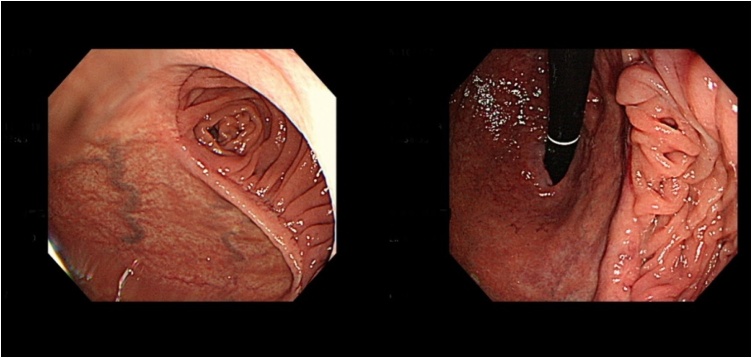
Esophagogastroduodenoscopy during follow-up, 6 months after discharge, showing no sign of recurrence.

The patient was discharged to home in good condition on postoperative day 7 after the second surgery, with hospital discharge instructions and an outpatient department appointment. Six months later, clinical follow-up with EGD showed no sign of recurrence (Fig. 7).

## 3 Discussion

The anatomical distribution of vascular supply and collateral vessels around the stomach make it an organ with exceptionally rich blood supply [2,3]; thus, necrosis in any part of the stomach is a rare complication post-gastrectomy. However, postoperative gastric remnant ischemia is a life-threatening condition in gastrectomy cases especially when accompanied by other comorbid diseases like atherosclerosis or venous thrombosis [5] or intra-operative complications such as bleeding or conditions requiring splenectomy [6].

In our case, wherein splenectomy was performed, although a subsequent Billroth-I reconstruction was executed based on approximately 50 % viable well-perfused gastric remnant, we still speculate that the long remnant part of the stomach after splenectomy and ligation of the short gastric vessels may have led to a significantly reduced perfusion to the distal part of the stomach.

Moreover, a major concern in these cases is that gastric anastomosis disruption and perforation due to ischemia may lead to a high risk of morbidity and mortality in up to 70 % of cases [7,8]; however, our patient did not show any clinical signs of peritonitis. Further, timely CT examination with contrast may help in the detection of ischemia, leakage, or perforation, although, CT sometimes cannot rule out the early signs of ischemia in the early postoperative period [9]. We consider EGD to be the most effective diagnostic modality used in this case that revealed necrotic patches covering the mucosal surface of distal remnant stomach albeit with intact anastomosis and duodenal mucosa, thus facilitating early diagnosis. Although some similar cases have been reported previously, our case was managed completely on the basis on the clinical findings of the patient [10]. Moreover, we selected a completely laparoscopic approach in contrast to the other reported cases wherein laparotomy was employed.

Close follow-up of the patient’s condition, including that for clinical, laboratory, and radiological findings, can facilitate the evaluation of the recovery and detection of worsening and progression of the necrosis. It can also help determine the appropriate time for a repeat EGD, which may be more informative in addition to the clinical parameters as in our case to determine the appropriate time for surgical intervention either in stable condition as a semi-elective procedure, as in our case, or in emergency situations if the condition deteriorates. Laparoscopic exploration and management in such cases may carry some risk of bleeding, leakage, and perforation of other intact parts due to adhesions, active inflammatory processes, and tissue friability, but is still a feasible and good option, as seen in our case, when performed by an expert surgeon.

## 4 Conclusion

Careful dissection of the lymph nodes over the major vessels is essential to avoid complications in gastric cancer patients requiring laparoscopic surgeries. Moreover, major complications occurring intra-operatively may necessitate changes in the surgical plan, including re-excision of the remnant stomach or conversion to Roux-en-Y reconstruction. Further, laparoscopic subtotal gastrectomy for remnant gastric ischemia after LDG is a feasible management approach and can help to minimize the short and long term post-operative complications and enhance the quality of life of patients. EGD is considered the cornerstone diagnostic tool for the detection of early signs of gastric remnant ischemia especially in the early postoperative period.

## Funding

This study did not receive any financial support.

## Ethical approval

This study is exempt from ethical approval at Asan Medical Center Cancer Institute. Patient approval and written consent was taken.

## Consent

Written informed consent was obtained from the patient for publication of this case report and accompanying images. A copy of the written consent is available for review by the Editor-in-Chief of this journal on request.

## Author contribution

All authors contributed equally in this study.

## Registration of research studies

1. Name of the registry: Laparoscopic management of gastric remnant ischemia after laparoscopic distal gastrectomy with Billroth-I anastomosis- A case report.

2. Unique Identifying number or registration ID: researchregistry5149

3. Hyperlink to the registration (must be publicly accessible): https://www.researchregistry.com/browse-the-registry#home/

## Guarantor

The guarantor for this study is

1) Abdulaziz Alshehri.

2) Kim Hee-sung.

3) Kim Byung Sik.

## Provenance and peer review

Not commissioned, externally peer-reviewed

## Declaration of Competing Interest

There is no conflicts of interest to declare by authors.
